# How to Rise

**Published:** 1864-03

**Authors:** 


### HOW TO RISE.

FRENCH statisticians say that the "well to do" live about eleven years longer, on an average, than those who work from day to day for a living, who, if they fail to get work to-day, will have no bread to eat to-morrow, unless they obtain it on credit, borrow, beg, or steal. Hence, it is clear that the moral debasements of the last two, and the wearing economies and anxieties attendant on the first named, tend to shorten human life. If, then, the young can have pointed out to them a sure means of rising in the world, of attaining a happy competence, it is the legitimate province of the physician to indicate what these means are, as applicable to a large class of readers. Let every young man and young woman bear in mind always, that their destiny in life depends on their individual character; that what that is they will inevitably be; because the character of a man is indicated by his actions. These actions are read by the observant, and they are "placed" accordingly. Hence, all should study propriety of deportment; not only as to the greater, but in regard to things which may be considered of scarcely any importance.

A shrewd young man, one who is destined to "rise in the world," would never select that girl for a wife who would sit down on a book which chanced to lie on a chair or sofa, who would tread on or over a pocket-handkerchief, or any article of clothing on the floor, rather than stoop to pick it up.

A young lady of taste and refinement would scarcely accept the attentions of a young man the collar of whose coat was usually speckled with dandruff, or whose finger-ends were fringed with black, or whose shirt-bosom was often spotted with tobacco-juice, or was uniformly ruffled or soiled.

Those who are so full of the milk of human kindness as to promise unhesitatingly almost any thing asked of them, and are just as full, later on, of excellent reasons for not having fulfilled these promises, can never make their way into the confidence and respect of the thrifty and the good.

A man of good repute in Wall street, the other day applied to a well-known citizen to rent from him a furnished house. He was refused. A mutual friend expressed surprise. "He stands well on the street." "Yes." "His family are highly esteemed." "Yes." "He is known to be punctual in all his pecuniary engagements." "Yes."

"Well, why won't you let him have your house, at your own price, while you are away?"

"Because he came into my parlor and sat on my sofa with his hat on. Such a man can not have habits of personal neatness. He would spit on my carpets; he would break my chair-backs by tilting them against the wall, and soil it with his unkempt hair. The presumption is, his family are like him; at all events, he alone could injure my furniture more in six months than would be the profits of renting. No, sir! A man who sits in my parlor with his hat on, the first time he ever entered it, can not rent my house at any price."

Each defect in a person's character is read by the observant as easily as the scarlet letter on the back of the erring. Let the young remember that the character will "crop out" in the manners, in the little acts of life, and that, if these are unexceptionable, and if they are uniformly neat, methodical, prompt, and energetic, these qualities will prove a passport to "good places," and to that thrift which brings with it a quiet mind and length of days.

